# ADAP1 limits neonatal cardiomyocyte hypertrophy by reducing integrin cell surface expression

**DOI:** 10.1038/s41598-018-31784-w

**Published:** 2018-09-11

**Authors:** Hugo Giguère, Audrey-Ann Dumont, Jonathan Berthiaume, Vanessa Oliveira, Gino Laberge, Mannix Auger-Messier

**Affiliations:** 1 Département de Pharmacologie et Physiologie, Faculté de Médecine et des Sciences de la Santé, Université de Sherbrooke, Sherbrooke, QC Canada; 2 Département de Biochimie, Faculté de Médecine et des Sciences de la Santé, Université de Sherbrooke, Sherbrooke, QC Canada; 3 Département de Médecine – Service de Cardiologie, Centre de Recherche du CHUS, Faculté de Médecine et des Sciences de la Santé, Université de Sherbrooke, Sherbrooke, QC Canada

## Abstract

The ArfGAP with dual PH domains 1 (ADAP1) regulates the activation of the hypertrophic mitogen-activated protein kinase ERK1/2 pathway in non-cardiomyocytes. However, its role in cardiomyocytes is unknown. Our aim was to characterize the role of ADAP1 in the hypertrophic process of cardiomyocytes. We assessed the expression of ADAP1 in the hearts of adult and neonatal rats by RT-qPCR and Western blotting and showed that it is preferentially expressed in cardiomyocytes. Adenoviral-mediated ADAP1 overexpression in cultured rat neonatal ventricular cardiomyocytes limited their serum-induced hypertrophic response as measured by immunofluorescence microscopy. Furthermore, ADAP1 overexpression completely blocked phenylephrine- and Mek1 constitutively active (Mek1ca) mutant-induced hypertrophy in these cells. The anti-hypertrophic effect of ADAP1 was not caused by a reduction in protein synthesis, interference with the Erk1/2 pathway, or disruption of the fetal gene program activation, as assessed by nascent protein labeling, Western blotting, and RT-qPCR, respectively. An analysis of cultured cardiomyocytes by confocal microscopy revealed that ADAP1 partially re-organizes α-actinin into dense puncta, a phenomenon that is synergized by Mek1ca overexpression. Biotin labeling of cell surface proteins from cardiomyocytes overexpressing ADAP1 revealed that it reduces the surface expression of β1-integrin, an effect that is strongly potentiated by Mek1ca overexpression. Our findings provide insights into the anti-hypertrophic function of ADAP1 in cardiomyocytes.

## Introduction

ArfGAP with dual PH domains 1 (ADAP1), also known as centaurin-α1, is a GTPase-activating protein (GAP) that regulates the activity of small GTPases from the ADP-ribosylation factor (ARF) family^1^. ADAP1 was first identified in the brain and the heart, but its role in cardiac function was not investigated at the time^2^. ADAP1 interacts with nucleolin, Kif13b, and protein kinase C^3–5^, and is also functionally linked with the activation of the mitogen-activated protein kinases (MAPKs) ERK1/2^6,7^, which is of particular interest in a cardiac context, especially since the MEK1-ERK1/2 pathway supports and potentiates cardiac hypertrophy^8–10^. This signaling pathway is activated in response to several stresses in the heart, including hypertrophic agonist stimulation and mechanical stretching via integrins^11,12^. On the other hand, inhibiting ERK1/2 blocks the onset of hypertrophy in many cases^13,14^. ADAP1 also regulates small G protein ARF6 activity through its GAP domain, catalyzing the conversion of active ARF6-GTP to inactive ARF6-GDP^1,15,16^. In turn, ARF6 regulates different processes that occur in cell membranes, such as vesicular trafficking (endocytosis), membrane fusion/budding, and cytoskeletal reorganization^17–19^.

Interestingly, ARF6 also regulates the surface expression and recycling of integrins in HeLa and endothelial cells^20,21^. Notably, Hongu *et al*. showed that ARF6 is required for hepatocyte growth factor-induced endothelial cell spreading and β1-integrin recycling^21^. Integrins do not simply act as inert anchors of cells to the extracellular matrix, they also play an active role in the hypertrophic process of cardiomyocytes^22,23^. Indeed, β1-integrin overexpression in cultured cardiomyocytes promotes cell hypertrophy^24^, while down-regulation of β1-integrin at the surface of cardiomyocytes impedes such hypertrophy^25^.

Based on these potential signaling mechanisms, we hypothesized that ADAP1 may regulate cardiomyocyte hypertrophy by interfering with integrin-dependent processes. We show for the first time that ADAP1 can block cardiomyocyte hypertrophy *in vitro* by reducing the expression of β1-integrin at the cell surface.

## Results

### Adap1 is expressed in cardiomyocytes

**Figure 1:**
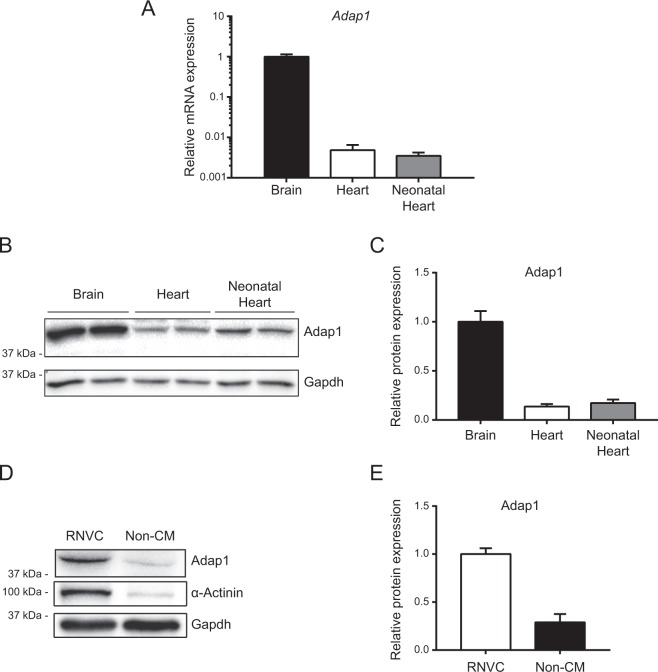
Adap1 expression in cardiac cells. (**A**) Relative levels of *Adap1* mRNA expression in the rat brain (adult) and heart (adult and 2-day-old neonate) were measured by RT-qPCR and were normalized to the *Rpl30* reporter gene (*n* = 4 independent tissues). (**B**) Representative Western blots of Adap1 and Gapdh (loading control) detected from whole brain and heart extracts. (**C**) The histogram represents the relative expression level of Adap1 normalized to Gapdh in the respective tissues (*n* = 4 independent tissues). (**D**) Representative Western blot of Adap1, α-Actinin (cardiomyocyte specific marker), and Gapdh (loading control) detected in the protein lysates of enriched rat neonatal ventricular cardiomyocytes (RNVC) and non-cardiomyocytes (Non-CM). (**E**) The histogram represents the relative expression level of Adap1 normalized to Gapdh in the respective cell lysates (*n* = 4 independent cell isolations). Full-length blots are presented in Supplementary Fig. 1.

When ADAP1 was first cloned, one clue suggested that it is expressed to some extent in the hearts of adult rats^2^. In order to validate this observation, we analyzed the cardiac expression of Adap1 in Sprague Dawley rats. Compared to the level of *Adap1* mRNA expression observed in the brain, *Adap1* mRNA transcripts were expressed at the relative levels of 0.5% and 0.35% in the hearts of adult and neonatal rats, respectively (Fig. 1A). The presence of an *Adap1* transcript in whole heart extracts was also confirmed by end-point PCR detection of putative splice variants that display the same unique transcript detected in whole brain extracts (data not shown). As expected, Western blotting analyses confirmed that whole brain lysates contained high levels of Adap1 (Fig. 1B). Interestingly, Adap1 was also detected in whole heart extracts of adult and neonatal rats at levels relative to the brain of 13% and 17%, respectively (Fig. 1B,C). A similar expression profile was also obtained using another antibody targeting a different immunogen sequence of Adap1 (data not shown). Since the heart is composed of different cells, including cardiomyocytes, endothelial cells, and fibroblasts^26^, we determined which of these cell types preferentially expressed Adap1. As measured by Western blotting, Adap1 was 3-fold more abundant in isolated rat neonatal ventricular cardiomyocytes (RNVC)

than in partially enriched non-cardiomyocyte (non-CM) cells (Fig. 1D,E), suggesting that it likely plays a role in cardiomyocytes.

### ADAP1 hinders the hypertrophy of cardiomyocytes

**Figure 2:**
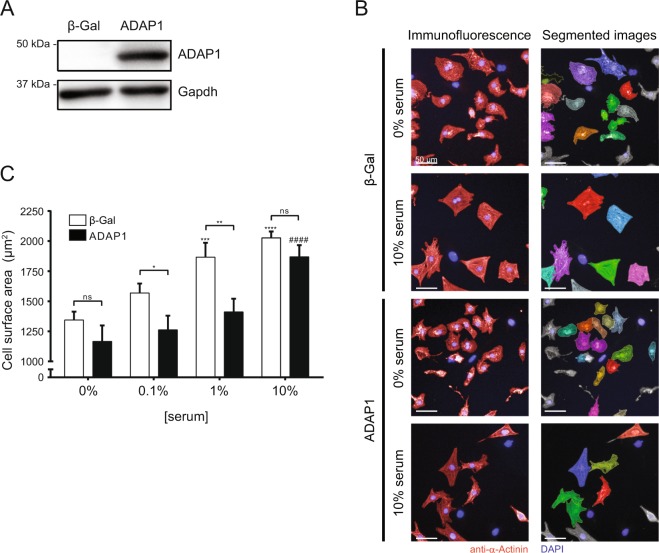
ADAP1 restrains the serum-induced increase in cell size of cultured cardiomyocytes. (**A**) Western blot detection of adenovirus-mediated 3xFLAG-hADAP1 overexpression (MOI of 50) in rat neonatal ventricular cardiomyocytes (RNVC) cultured for 72 h post-infection. (**B**) RNVC were infected with either β-Gal- (negative control) or ADAP1-overexpressing adenovirus and were cultured for 72 h in the absence (0%) or presence (10%) of serum. Representative images of α-Actinin-immunostained RNVC (left) and corresponding segmented images were acquired using the Operetta High-Content Imaging System (Perkin Elmer). The scale bar represents 50 µm. (**C**) The histogram represents the cell surface areas of RNVC overexpressing either β-Gal or ADAP1 and cultured for 72 h with increasing concentrations of serum (*n* = 3 independent experiments). *P < 0.05 and **P < 0.005 vs. β-Gal; ***P < 0.0005 and ****P < 0.0001 vs. β-Gal at 0% [serum]. ^####^*P* < 0.0001 vs. ADAP1 at 0% [serum]; ns, not significant; Two-way ANOVA with Sidak’s multiple comparison test. Full-length blots are presented in Supplementary Fig. 2.

The overexpression of ADAP1 affects the cytoskeletal organization of epithelial HeLa cells and increases dendritic filopodia formation by primary cultured hippocampal neurons^1,16^. Since the reorganization of the cytoskeleton of cultured cardiomyocytes mediates their hypertrophic response to external stimuli^27^, we hypothesized that ADAP1 overexpression might affect the serum-induced increase in size of RNVC. Western blotting analyses confirmed that efficient adenoviral-mediated overexpression of ADAP1 occurs in cultured RNVC (Fig. 2A). We imaged α-actinin-immunostained RNVC and measured their cell surface area (highlighted as segmented images in Fig. 2B) following a 72-h incubation with increasing serum concentrations. In the absence of serum, RNVC overexpressing ADAP1 tended to be smaller than control cells infected with adβ-Gal, although the difference was not significant (Fig. 2B,C). While the size of adβ-Gal-infected RNVC increased significantly and dose-dependently in response to serum, ADAP1 limited the hypertrophic response of RNVC to this external stimulus. In fact, ADAP1 prevented any significant increase in the cell surface area of RNVC cultured with 0.1% and 1% serum. However, 10% serum was sufficient to increase the cell size of ADAP1-overexpressing RNVC to approximately the same extent as control cells (Fig. 2B,C). Although the overexpression of ADAP1 did not significantly change the serum-induced regulation of *Myh6* and *Myh7* mRNA expression, it delayed the upregulation of *Nppa* transcript level in response to increasing serum concentration (data not shown) and corroborated with the corresponding cell size.

**Figure 3:**
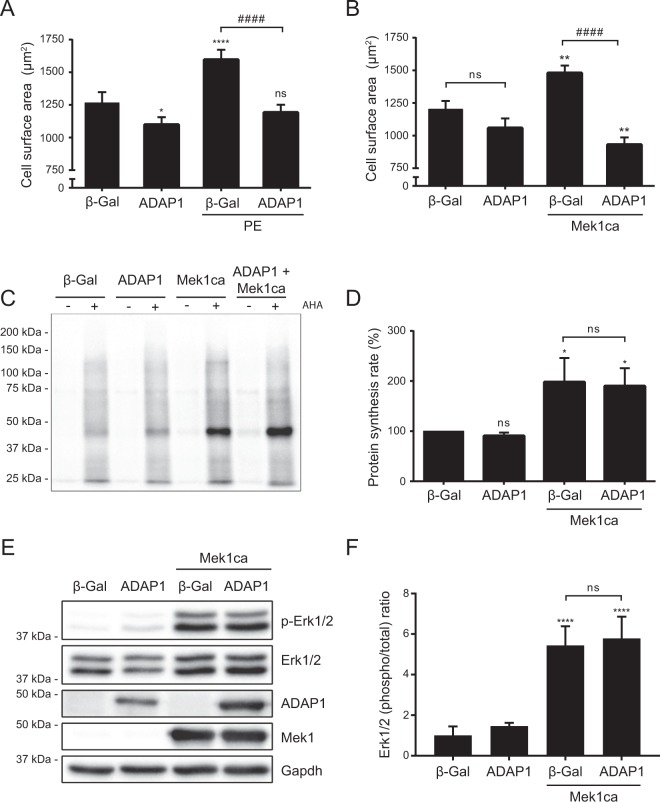
ADAP1 blocks phenylephrine- and Mek1ca-induced hypertrophy. Cell surface area measurements of rat neonatal ventricular cardiomyocytes (RNVC) overexpressing ADAP1 in the absence or presence of 50 µM phenylephrine (**A**) or Mek1ca-overexpressing adenovirus (**B**) compared with an adβ-Gal-infected control. Quantification of at least 3 independent experiments expressed as means ± SD. *P < 0.05; **P < 0.005; ****P < 0.0001 vs. unstimulated β-Gal. ^####^*P* < 0,0001 vs. stimulated β-Gal; ns, not significant; One-way ANOVA with Tukey’s multiple comparison test. (**C**) Representative Western blots of protein lysates from adβ-Gal-, adADAP1-, or adMek1ca-infected RNVC metabolically labeled for 2 h with L-azidohomoalanine (AHA) and detected with biotin-alkyne and streptavidin-HRP. (**D**) The histogram represents the rates of nascent protein synthesis relative to the β-Gal control, which were measured using the same experimental conditions as in **C** (*n* = 3 independent experiments). *P < 0.05 vs. β-Gal; ns, not significant; One-way ANOVA with Tukey’s multiple comparison test. (**E**) Representative Western blots of Erk1/2 phosphorylation status, ADAP1 and Mek1ca overexpression levels, and Gapdh (loading control) from protein lysates of RNVC cultured for 72 h following the indicated adenoviral infection. (**F**) The histogram represents the phospho/total Erk1/2 ratios relative to the β-Gal control and which were measured using the same experimental conditions as in **E** (*n* = 4 independent experiments). ****P < 0.0001 vs. β-Gal; ns, not significant; One-way ANOVA with Tukey’s multiple comparison test. Full-length blots are presented in Supplementary Fig. 3.

The serum and phenylephrine pro-hypertrophic stimulus do not regulate the expression of endogenous Adap1 at the transcriptional and translational levels in RNVC (data not shown). Interestingly, the overexpression of ADAP1 completely blocked the phenylephrine-induced hypertrophy of RNVC (Fig. 3A). Since both serum- and phenylephrine-dependent responses in cardiomyocytes commonly converge toward the hypertrophic MEK1-ERK1/2 signaling pathway^10^, we investigated how ADAP1 would impact the effect on RNVC cell size of overexpressing a Mek1 constitutively active mutant (Mek1ca). ADAP1 not only completely blocked the Mek1ca-induced hypertrophy of RNVC, it significantly reduced their size even further compared to adβ-Gal-infected control cells (Fig. 3B). However, the 2-fold acceleration in the rate of Mek1ca-induced protein synthesis (Fig. 3C,D) and the increase in Erk1/2 phosphorylation (Fig. 3E,F) were not inhibited nor altered by the overexpression of ADAP1, suggesting that ADAP1 potently impedes other components of the hypertrophic process in cardiomyocytes.

### ADAP1 does not interfere with the fetal gene program

**Figure 4:**
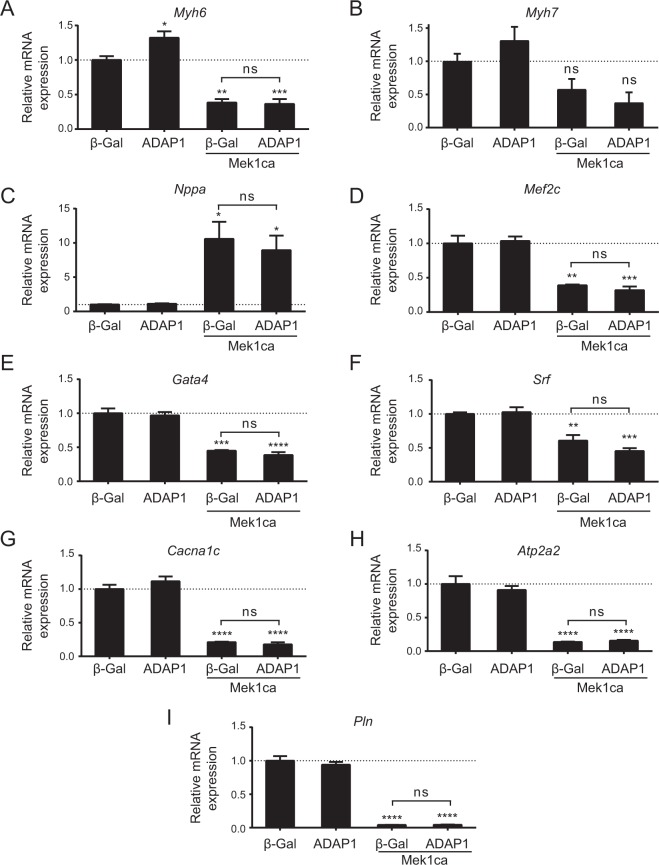
ADAP1 does not interfere with Mek1ca-induced fetal gene program activation. (**A**–**I**) Analysis by RT-qPCR of different mRNA expressed in rat neonatal ventricular cardiomyocytes (RNVC) that are representative of the fetal gene program. The RNVC were infected with an β-Gal- (negative control), ADAP1-, or Mek1ca-overexpressing adenovirus, individually or in combination as indicated, and were cultured for 72 h post-infection. The histograms represent mRNA expression levels relative to the β-Gal control and normalized to the *Rpl30* reporter gene (*n* = 3 independent experiments). *P < 0.05; **P < 0.005; ***P < 0.0005; ****P < 0.0001 vs. β-Gal; ns, not significant; One-way ANOVA with Tukey’s multiple comparison test.

The hypertrophic response of cardiomyocytes is often associated with the re-expression or repression of genes corresponding to the fetal stage of cardiac development^28,29^. While Mek1ca overexpression in RNVC triggers the fetal gene program, the activation of the program, as well as cardiomyocyte hypertrophy, are abrogated by the genetic or pharmacological inhibition of the MEK1-ERK1/2 pathway^30–33^. In our experimental conditions, the infection of RNVC with adMek1ca provokes a 60% decrease in *Myh6*, *Mef2c*, and *Gata4* mRNA expression (Fig. 4A,D,E) and a 40% decrease in *Srf* (Fig. 4F) mRNA expression. The *Myh7* gene was not significantly affected by Mek1ca overexpression (Fig. 4B) while the expression of the *Nppa* gene was increased over 10-fold (Fig. 4C). In addition, the expression of the calcium signaling-related genes *Cacna1c*, *Atp2a2*, and *Pln* was reduced by more than 75% in the presence of Mek1ca (Fig. 4G–I). More importantly, the co-expression of ADAP1 and Mek1ca did not significantly affect any of these changes in gene expression compared to the overexpression of Mek1ca alone. These results suggest that the anti-hypertrophic effect of ADAP1 is dominant over Mek1ca-mediated transcriptional regulation.

### ADAP1 regulates α-actinin organization and β1-integrin cell surface expression

**Figure 5:**
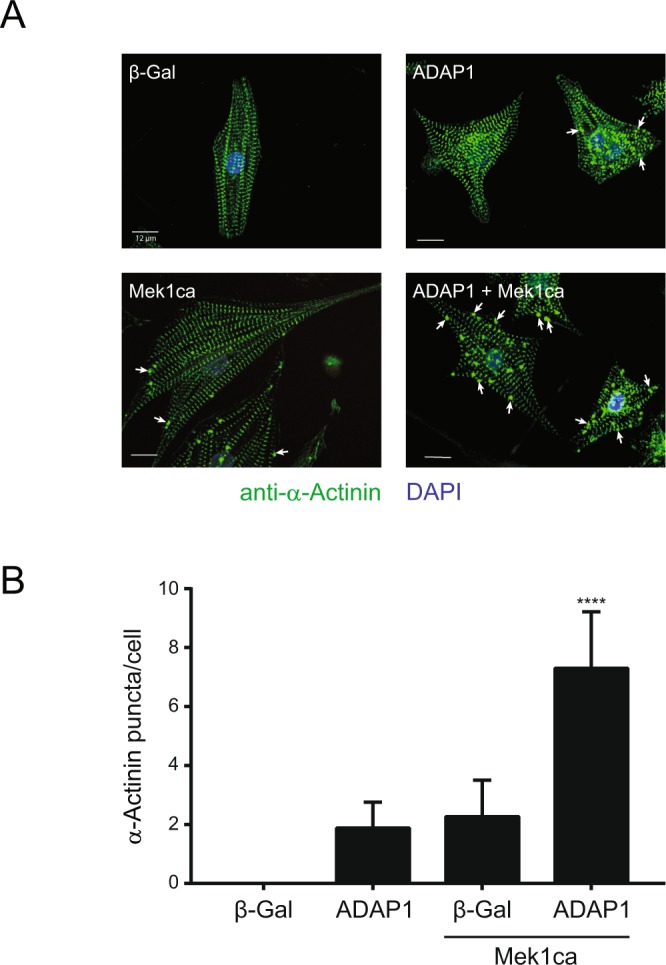
ADAP1 relocalizes cytoskeletal α-Actinin. (**A**) Representative confocal images (Olympus FluoView FV1000 microscope) of α-Actinin-immunostained rat neonatal ventricular cardiomyocytes (RNVC) infected with a β-Gal- (negative control), ADAP1-, or Mek1ca-overexpressing adenovirus, individually or in combination as indicated, and cultured for 72 h post-infection. Arrows point to α-Actinin dense puncta. The scale bar represents 12 µm. (**B**) Number of α-Actinin puncta per cell measured with the Operetta High-Content Imaging System (Perkin Elmer) using the same experimental conditions as in **A** (*n* = 4 independent experiments). ****P < 0.0001 vs. β-Gal; One-way ANOVA with Tukey’s multiple comparison test.

A careful examination of immunostained RNVC revealed that the overexpression of both ADAP1 and Mek1ca have a significantly greater impact on α-actinin organization than in control adβ-Gal-infected RNVC. Indeed, higher definition imaging by confocal microscopy revealed that the combined overexpression of ADAP1 and Mek1ca in RNVC induces the formation of multiple α-actinin dense puncta (Fig. 5A). Interestingly, ADAP1 or Mek1ca alone also induced, to some extent, the formation of such large, distinct cytoskeletal structures while control adβ-Gal-infected RNVC did not (Fig. 5A). Increasing the overexpression of ADAP1 up to a multiplicity of infection (MOI) of 100 did not further increase the number of α-actinin puncta (data not shown). More importantly, quantifying the number of these α-actinin puncta per cell using the Operetta High-Content Imaging System revealed that the combined overexpression of ADAP1 and Mek1ca has a synergistic effect in RNVC (Fig. 5B).

**Figure 6:**
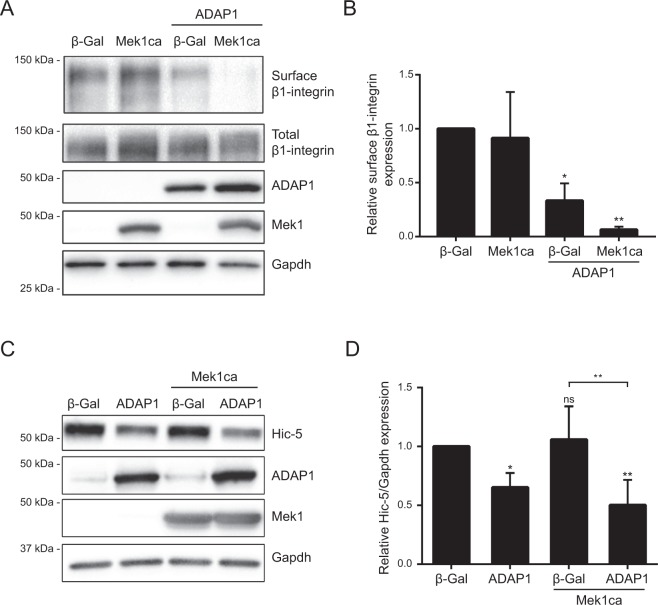
ADAP1 reduces cell surface β1-integrin expression in cardiomyocytes. (**A**) Representative Western blot of biotin-labeled cell surface β1-integrin expression compared to total β1-integrin expression in protein lysates of rat neonatal ventricular cardiomyocytes (RNVC) infected with a β-Gal- (negative control), ADAP1-, or Mek1ca-overexpressing adenovirus, individually or in combination as indicated, and cultured for 72 h post-infection. The expression levels of ADAP1, Mek1ca, and Gapdh (loading control) were also verified. (**B**) The histogram represents the level of cell surface β1-integrin expression normalized to total β1-integrin expression relative to the β-Gal control and measured using the same experimental conditions as in **A** (*n* = 3 independent experiments). *P < 0.05; **P < 0.005 vs. β-Gal; One-way ANOVA with Tukey’s multiple comparison test. (**C**) Representative Western blot of Hic-5, ADAP1, Mek1ca, and Gapdh (loading control) in protein lysates obtained from RNVC cultured for 72 h following the indicated adenoviral infection. (**D**) The histogram represents the relative expression levels of Hic-5 normalized to Gapdh, relative to the β-Gal control, and measured under the same experimental conditions as in **C** (*n* = 4 independent experiments). *P < 0.05; **P < 0.01 vs. β-Gal; ns, not significant; One-way ANOVA with Fisher’s LSD test. Full-length blots are presented in Supplementary Fig. 6.

Given that the activation of the Mek1-Erk1/2 signaling pathway in RNVC increases the expression of integrins and modulates their localization at the cell surface, promoting hypertrophy^34^, we hypothesized that ADAP1 may interfere with these processes. Indeed, cell surface biotin labeling indicated that ADAP1 by itself significantly reduces the localization of β1-integrin at the surface of RNVC without changing the total expression of β1-integrin (Fig. 6A,B). Strikingly, β1-integrin was almost completely absent from the surface of RNVC overexpressing both ADAP1 and Mek1ca (Fig. 6A,B), indicating that ADAP1 impairs the processes required for the normal expression of β1-integrin at the surface of RNVC.

Among the β1-integrin-associated proteins forming the cardiac costameres, it is interesting to note that the adaptor protein Hic-5, a paxillin family member, is essential for the hypertrophic process of cultured RNVC^35^. To gain a mechanistic insight into the ADAP1 anti-hypertrophic effect, we verified how ADAP1 impacts Hic-5 expression in RNVC. The overexpression of ADAP1, alone or combined with Mek1ca, reduced Hic-5 expression levels to approximately 65% and 50%, respectively, compared to control adβ-Gal-infected RNVC (Fig. 6C,D). Altogether, these results suggest that ADAP1 interferes with the formation of these signaling hubs, which are required for cardiomyocyte hypertrophy.

## Discussion

In the present study, we identified and characterized, for the first time, the role of Adap1 in cardiomyocytes. We showed that (i) Adap1 is present in both neonatal and adult rat hearts and that it is predominantly expressed in cardiomyocytes, (ii) the overexpression of ADAP1 restrains the development of serum-induced hypertrophy and completely blocks Mek1ca-induced hypertrophy in cultured RNVC, and (iii) ADAP1 reduces the localization of β1-integrin at the cardiomyocyte surface and, as such, the hypertrophic process of cardiomyocytes.

*Adap1* mRNA and protein are expressed by a variety of neurons, including cortical, cerebellar, and hippocampal neurons, with peak mRNA expression reached between two and four weeks postnatally in rats^36^. While Adap1 is highly expressed in the whole brain, little is known about its expression in other tissues^2,36,37^. While a few indications suggest that Adap1 may be expressed in the heart, this possibility has not been verified or studied in detail. For instance, a protein expressed in both the brain and heart tissues of adult rats was detected by photoaffinity labeling with an inositol 4-phosphate analog, which led to the cloning of Adap1^2^. The Human Protein Atlas also indicates that ADAP1 has been detected by immunohistochemistry in the cardiomyocytes of adult heart specimens^38^.

To further validate these observations, we showed that Adap1 is indeed expressed at similar levels in the hearts of neonatal and adult rats. As previously reported^2,36,37^, we showed that the *Adap1* transcript is highly expressed in the brain of adult rats but is much less abundant in other tissues such as the heart. Nonetheless, we easily detected the Adap1 protein by Western blotting in whole heart extracts, suggesting that factors other than low transcript levels may mediate the high expression in cardiac tissue. This would be in agreement with the significantly slower turnover rate and longer metabolic half-life (t½ = 6.93 days) of Adap1 reported in primary cultures of cortical neurons^39^. Like the unique *Adap1* transcript variant detected in both brain and heart extracts by end-point PCR, the band of Adap1 protein expressed in these tissues migrated at the same molecular weight (43 kDa) previously reported for Adap1^2,40^. Since Adap1 was more abundantly expressed in isolated RNVC than in partially enriched non-cardiomyocytes cells, these findings suggest that it might contribute to cardiomyocyte development or function.

Adap1 functionally interacts with MAPKs Erk1/2 to activate this signaling pathway in COS-7 cells and hippocampal neurons through mechanisms that are not fully understood^6,7,41^. Since increased signaling of the Mek1-Erk1/2 axis induces cardiomyocyte hypertrophy^9,30,42^, we hypothesized that ADAP1 might significantly promote the hypertrophic process in these cells. To verify this hypothesis, we overexpressed ADAP1 in RNVC but did not observe a significant increase in Erk1/2 phosphorylation under basal conditions. In addition, an acute stimulation with phenylephrine, a hypertrophic agonist, activated Erk1/2 more efficiently in RNVC overexpressing ADAP1 (data not shown). However, rather than increasing the size of cardiomyocytes in the absence of serum, ADAP1 caused a slight reduction in their cell surface area compared to adβ-Gal-infected control cells. Although an anti-hypertrophic effect was also observed at lower serum concentrations (0.1 and 1%), RNVC overexpressing ADAP1 were still able to hypertrophy in response to a higher concentration of serum (10%). This may be explained by the fact that serum contains several active factors able to induce cardiomyocyte hypertrophy through different Erk1/2-dependent and -independent signaling pathways^43–45^.

We thus overexpressed the hypertrophic Mek1ca mutant in RNVC to determine whether sustained specific Erk1/2 activation could overcome the anti-hypertrophic effect of ADAP1. As expected, Mek1ca increased the cell size and protein synthesis rate of RNVC, which are hallmarks of cardiomyocyte hypertrophy, in our culture conditions. Interestingly, its co-expression with ADAP1 further reduced the cell surface area of RNVC to a significantly smaller size than that of adβ-Gal-infected control cells. The dominant effect of ADAP1 over Mek1ca-induced cardiomyocyte hypertrophy was not due to the normalization of the protein synthesis rate nor to Erk1/2 phosphorylation, which would have been surprising given the propensity of ADAP1 to favor Erk1/2 signaling. In addition, ADAP1 did not interfere with the Mek1ca-induced activation of the fetal gene program, another hallmark of *in vitro* and *in vivo* cardiomyocyte hypertrophy and which is regulated in part by Erk1/2 signaling^28,29,46,47^. Compared to previous reports^10,30,48^, the broader transcriptional profile analysis performed in the current study is the first, to our knowledge, to examine more broadly the particular Mek1ca-induced fetal gene program in cultured rat neonatal ventricular cardiomyocytes. Taken together, these results suggest that ADAP1 interferes with an Erk1/2-independent mechanism to block Mek1ca-induced hypertrophy in RNVC.

Since ADAP1 overexpression modifies cytoskeleton organization in other cell types^1,49^, we determined, by confocal microscopy, whether it has a similar effect in cardiomyocytes. The overexpression of ADAP1 significantly increased the formation of α-actinin dense puncta in RNVC. Strikingly, the co-expression of ADAP1 and Mek1ca synergistically increased the number of α-actinin puncta per cell, suggesting that both signaling molecules are involved in a common cytoskeletal reorganization mechanism. In addition to being a major cytoskeletal component of the sarcomeric Z-line, α-actinin also links the sarcomere with the extracellular matrix through integrins^50^. This prompted us to determine whether ADAP1 affects the expression of β1-integrin at the cardiomyocyte surface since these integral membrane proteins regulate processes involved in cardiac hypertrophy^21–23^. Indeed, ADAP1 significantly decreased the expression of β1-integrin in the sarcoplasmic membrane while its co-expression with Mek1ca almost completely eliminated its expression at the surface of cardiomyocyte.

Cardiac costamer components such as paxillin also influence the internalization/recycling of integrin and the cardiomyocyte hypertrophic process^51^. For instance, the adaptor protein Hic-5, a paxillin family member, is essential for phenylephrine-induced hypertrophy of cultured RNVC^35^. We thus hypothesized that ADAP1 may interfere with Hic-5. We showed that the overexpression of ADAP1, alone or together with Mek1ca, significantly reduces Hic-5 protein expression in RNVC. Although Mek1ca-induced Erk1/2 phosphorylation seemed to be unaffected by the overexpression of ADAP1 in RNVC, we cannot exclude the possibility that the localization of activated Erk1/2 in specific subcellular compartments may have changed under these conditions, leading to an enhancement of α-actinin puncta formation and a reduction of Hic-5 expression and β1-integrin levels on the cell surface.

These results are in agreement with the opposing effects exerted by different Arf GAPs with regard to integrin signaling, internalization, and recycling^52^. For example, GIT1 and ACAP1 regulate the signaling of integrins in different cell types^53–55^. PAG3, another centaurin family member, interacts with paxillin and inhibits its recruitment at the integrin-rich focal adhesion complex^56^. Interestingly, after stimulating RNVC with the hypertrophic agonist endothelin-1, paxillin forms puncta structures that are similar to those observed in the present study^57^. The scaffold protein IQGAP1 also recruits melusin and focal adhesion kinase at a multiprotein complex required for Erk1/2-mediated cardiomyocyte hypertrophy^58^. Lastly, another interesting possibility arose from the demonstration that ADAP1 overexpression in HEK293 cells inhibits both basal and agonist-induced internalization of β2-adrenergic receptors^15^. While integrin internalization/recycling processes are different than G protein-coupled receptor trafficking, it would be interesting to determine whether Adap1 also regulates the expression of ion channels and proteins such as GPCRs at the surface of cardiomyocytes.

Collectively, these results provide new insights into the regulation of *in vitro* cardiomyocyte hypertrophy by Adap1 via the regulation of β1-integrin complexes. *In vivo* studies will be needed to further characterize the anti-hypertrophic function of Adap1 in cardiomyocytes exposed to physiological and pathological stresses.

## Methods

### Ethics

All animal procedures complied with NIH guidelines and were approved by the Université de Sherbrooke Institutional Committee for the Use and Care of Laboratory Animals.

### Adenovirus generation

**]**The adenoviruses encoding the β-galactosidase (β-Gal) and Mek1 constitutively active mutant (Mek1ca) were generous gifts from Jeffery D. Molkentin (HHMI, Cincinnati, OH, USA). The seamless assembly and subcloning of 3xFLAG-tagged hADAP1 cDNA (Cat. #11033; DF/HCC DNA Resource Core) into the Gateway pENTR3C Dual Selection vector (Invitrogen) was performed with a NEBuilder HiFi DNA Assembly Cloning kit (New England Biolabs). The recombination with the Gateway pAd/CMV/V5-DEST adenovirus vector (Invitrogen) was performed using the Gateway LR Clonase II Enzyme Mix (Invitrogen). After linearizing the vector with PacI (New England Biolabs), 4 µg of digested product was transfected into 90–100% confluent HEK 293 A cells grown in 35-mm dishes using Lipofectamine 2000 transfection reagent (Invitrogen). After 6–8 days of culture, the adenovirus was collected from lysed cells and was amplified by transferring 100 µL of the primary stock to a 10-cm dish of 90–100% confluent HEK 293 A cells. The cells and supernatant were collected 48–72 h later, freeze-thawed three times, and centrifuged at 100 RCF for 5 min to remove cell debris. The supernatant was collected, aliquoted, and stored at –80 °C. Serial dilutions of each adenovirus were used to infect HEK 293 A cells and determine their respective titers. Hexon-expressing cells immunostained using anti-hexon antibody (ab8249; Abcam) were quantified 36 h post-infection.

### Rat neonatal ventricular cardiomyocyte isolation and culture

RNVC were extracted from the hearts of Sprague Dawley rat pups (strain code 001; Charles River) 1–3 days after birth using the Neonatal Cardiomyocyte Isolation System (Worthington Biochemical Corporation). After decapitation, the hearts were removed, the atria were discarded, and the ventricles were cut in half. The isolated ventricles were incubated in calcium/magnesium-free Hank’s Balanced Salt Solution containing trypsin (50 µg/mL) in vented-cap tubes with slow agitation at 4 °C for 18 h. The trypsin was then inhibited by adding Soybean Trypsin Inhibitor (200 µg/mL), and the ventricles were further digested with collagenase (100 units/mL) at 37 °C for 30 min. Following trituration and filtration through a 70-micron cell strainer, the cells were pre-plated on 10-cm dishes at 37 °C for 30 min to reduce non-cardiomyocyte contamination. Enriched RNVC were resuspended in M199 medium containing 10% fetal bovine serum (FBS), 2 mM glutamine, 100 U/mL of penicillin, and 100 µg/mL of streptomycin. They were then plated at a density of 43,000 cells/cm^2^ on fibronectin-coated microplates for immunofluorescence or on gelatin-coated cell-culture dishes for other experiments. Five hours after plating, the RNVC were infected with the appropriate adenoviruses at an MOI of 50 and were cultured in a humidified incubator with 5% CO_2_ at 37 °C for 72 h before analysis.

### Real-time PCR

Total RNA was extracted from RNVC using RNeasy Mini kits (Qiagen) according to the manufacturer’s protocol. ProtoScript II Reverse Transcriptase (New England Biolabs) was used to prepare cDNA from 1000 ng of total RNA in 20 µL. Real-time PCR was performed on technical duplicates with cDNA diluted 30x in nuclease-free water supplemented with SsoAdvanced Universal SYBR Green Supermix (Bio-Rad) using a Mastercycler ep RealPlex (Eppendorf). Data were normalized to *Rpl30* and fold changes were obtained using the 2^(−ΔΔ*C*t)^ method^59^. Primer sequences are given in Table 1.

**Table 1:** Sequences of primers of target genes used for RT-qPCR.

Gene name	Gene description	Sequences (5′->3′)
*Rpl30*	Ribosomal protein L30	Fwd: TCTTGGCGTCTGATCTTGGT
		Rev: AAGTTGGAGCCGAGAGTTGA
*Adap1*	ArfGAP with dual PH domains 1	Fwd: CAAAGACCCTCTGGATGCCTT
		Rev: GGTGACTCTGGGTTGACGG
*Myh6*	Myosin heavy chain 6	Fwd: GGCCAAGAGCCGTGACATT
		Rev: TTGTGGGATAGCAACAGCGAG
*Myh7*	Myosin heavy chain 7	Fwd: CAACCTGTCCAAGTTCCGCA
		Rev: GGCATCCTTAGGGTTGGGTAG
*Nppa*	Natriuretic peptide A	Fwd: CGGCACTTAGCTCCCTCTCT
		Rev: GTTGCAGCCTAGTCCGCTCT
*Mef2c*	Myocyte-specific enhancer factor 2 C	Fwd: CCAAATCTCCTCCCCCTATGAATC
		Rev: GCTGACGGATATCCTCCCATT
*Gata4*	GATA binding protein 4	Fwd: CTGTGCCAACTGCCAGACTA
		Rev: AGATTCTTGGGCTTCCGTTT
*Srf*	Serum response factor	Fwd: CCGTGCTCAATGCCTTCTCT
		Rev: CGTCCAAGTTCACCACCTGTAG
*Atp2a2*	ATPase Sarcoplasmic/Endoplasmic Reticulum Ca2 + Transporting 2	Fwd: AATCTGGTGACGGATGGTCTG
		Rev: TTCGAAGTCTGGGTTGTCCTC
*Cacna1c*	Calcium voltage-gated channel subunit alpha1 C	Fwd: AGCAACTTCCCTCAGACGTTTG
		Rev: CACAGTGCTGACCGTGCTG
*Pln*	Phospholamban	Fwd: TACCTTACTCGCTCGGCTATCAG
		Rev: GACCTTCACGACGATGTCCAG

### Western blotting analysis

Proteins were extracted from cultured RNVC using RIPA lysis buffer containing 50 mM Tris-HCl (pH 7.4), 150 mM NaCl, 1% Triton X-100, 1% sodium deoxycholate, 0.1% SDS, 1 mM DTT, 5 mM EDTA, and Halt Protease and Phosphatase Inhibitor Cocktail (Thermo Fisher Scientific). Protein concentrations were determined using DC Protein Assay kits (Bio-Rad) according to the manufacturer’s protocol. Lysates with equal amounts of proteins were run on SDS-PAGE gels and were transferred to polyvinylidene difluoride (Immobilon PVDF; EMD Millipore) or nitrocellulose (Perkin Elmer) membranes. Rabbit anti-ADAP1 antibody (ABS179) was purchased from EMD Millipore. Another antibody directed against a different immunogen sequence of ADAP1 (hpa012049) was also tested (data not shown) and was purchased, together with the mouse monoclonal anti-α-actinin antibody (A7811; clone EA-53) from Sigma. Rabbit antibodies directed against GAPDH (5174), ERK1/2 (4695), phospho-ERK1/2 (4370), and MEK1/2 (9122) were purchased from Cell Signaling Technology. Rabbit anti-β1-integrin antibody (sc-8978) was purchased from Santa Cruz Biotechnology. Mouse anti-Hic-5 antibody (611164) was purchased from BD Biosciences. Secondary anti-rabbit-HRP antibody (7074) was purchased from Cell Signaling Technology. Signals were revealed using the Luminata Western HRP Crescendo ECL Substrate purchased from EMD Millipore. Western blot acquisitions and image analyses were performed using a ChemiDoc MP station and Image Lab software (Bio-Rad), respectively.

### Protein synthesis assay

Infected RNVC were serum-starved for 48 h and were cultured in methionine-free medium for 1 h prior to performing the assay. The culture medium was then replaced with medium containing 25 µM AHA (Click Chemistry Tools) for 2 h to allow the incorporation of this methionine analog into newly synthesized proteins. Cells were collected in RIPA lysis buffer and equal amount of proteins were labeled with biotin-alkyne according to the manufacturer’s protocol (Click Chemistry Tools). Briefly, the following reagents were added to the protein samples at the specified final concentration: 20 µM Biotin-Alkyne (Click Chemistry Tools), 2 mM CuSO_4_ (Bio Basic), 15 mM ascorbic acid (Fisher Bioreagents), and 5 mM THPTA (Sigma-Aldrich). The samples were incubated for 30 min in the dark and were then boiled in Laemmli buffer for 5 min. Equal amounts of proteins were loaded on SDS-PAGE gels and were analyzed for total biotinylation levels by Western blotting using a streptavidin-HRP conjugate.

### Immunofluorescence analysis

RNVC were fixed using 4% paraformaldehyde after 72 h of culture in 96-well imaging microplates (Corning) for cell surface area measurements. RNVC were permeabilized with Triton X-100 and were immunostained with anti-α-actinin primary antibody (cardiomyocyte specific marker, A7811; Sigma) and Alexa Fluor conjugated secondary antibody (4408; Cell Signaling Technology). Images were acquired and analyzed using the Operetta High-Content Imaging System (Perkin Elmer) with a 20X objective. Cell surface areas (in µm^2^) were measured from technical duplicates (10 fields per well) and were averaged using at least 400 cells per condition. For some experiments, fixed RNVC were also imaged with an Olympus FluoView FV1000 confocal microscope (40X objective; Olympus FV10 ASW 4.0 Viewer analysis software).

### Cell surface β1-integrin expression

RNVC were cultured for 72 h post-infection and were washed 3 times with ice-cold PBS (pH 8.0). Cell surface proteins were biotin-labeled at 4 °C for 1 h using the cell-impermeable reagent sulfo-NHS-biotin at a concentration of 0.5 mg/mL in PBS (pH 8.0). The cells were then washed once with ice-cold PBS containing 100 mM glycine for 10 min, followed by 2 washes with ice-cold PBS. The cells were collected in RIPA lysis buffer, and equal amounts of proteins (600 µg) were incubated with MagReSyn Streptavidin microsphere beads (ReSyn Biosciences) for 2 h at 4 °C. The beads were washed 3 times with wash buffer (80 mM sodium phosphate, 150 mM NaCl, pH 7.5), and were eluted by boiling in biotin-saturated Laemmli buffer for 10 min. Equal volumes of eluted proteins were loaded on an SDS-PAGE gel and were analyzed for cell surface β1-integrin expression by Western blotting as described above.

### Statistical analysis

All experiments were performed using at least 3 independent cell isolations. Statistical analyses were performed using GraphPad Prism 7 (GraphPad Software). One-way ANOVA with Tukey’s multiple comparison test or two-way ANOVA with Sidak’s multiple comparison test were used for hypothesis testing when appropriate. Quantitative data are expressed as means ± standard deviation, and differences with a *P* value < 0.05 were considered significant.

## Electronic supplementary material

Supplementary figures
