# Supplementary material for: ADAP1 limits neonatal cardiomyocyte hypertrophy by reducing integrin cell surface expression
Source: Sci Rep. 2018 Sep 11;8:13605. doi: 10.1038/s41598-018-31784-w (PMC6134004; doi:10.1038/s41598-018-31784-w)
Supplement: Supplementary file 1 — Supplementary figures [file 41598_2018_31784_MOESM1_ESM.pdf]

Supplementary Information

ADAP1 limits neonatal cardiomyocyte hypertrophy by reducing integrin cell surface expression

Hugo Giguère[1], Audrey-Ann Dumont[2], Jonathan Berthiaume[1], Vanessa Oliveira[3], Gino Laberge[3], and Mannix Auger-Messier[1,2,3]*

[1]Département de Pharmacologie et Physiologie, Faculté de Médecine et des Sciences de la Santé, Université de Sherbrooke, QC, Canada

[2]Département de Biochimie, Faculté de Médecine et des Sciences de la Santé, Université de Sherbrooke, QC, Canada

[3]Département de Médecine – Service de Cardiologie, Centre de Recherche du CHUS, Faculté de Médecine et des Sciences de la Santé, Université de Sherbrooke, Sherbrooke, QC, Canada

* Corresponding author: Mannix.Auger-Messier@USherbrooke.ca

Supplementary Figure S1

B

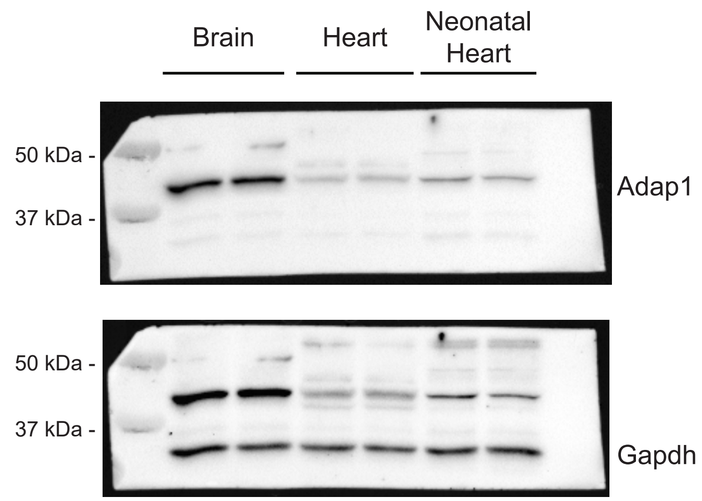

D

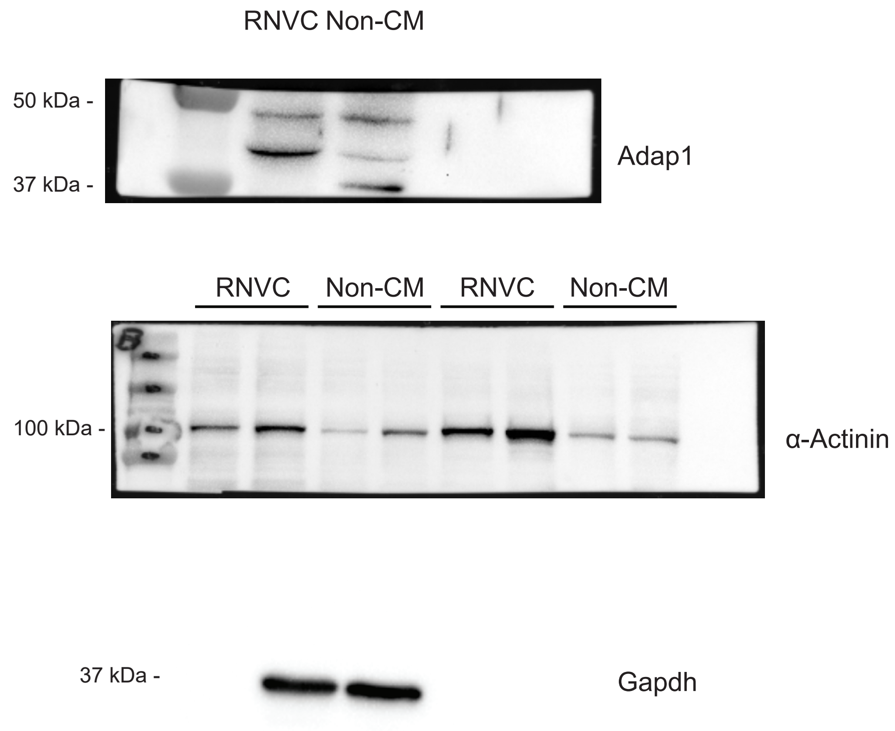

Supplementary Figure S1. ADAP1, Gapdh and α-Actinin full-length immunoblots

Supplementary Figure S2

A

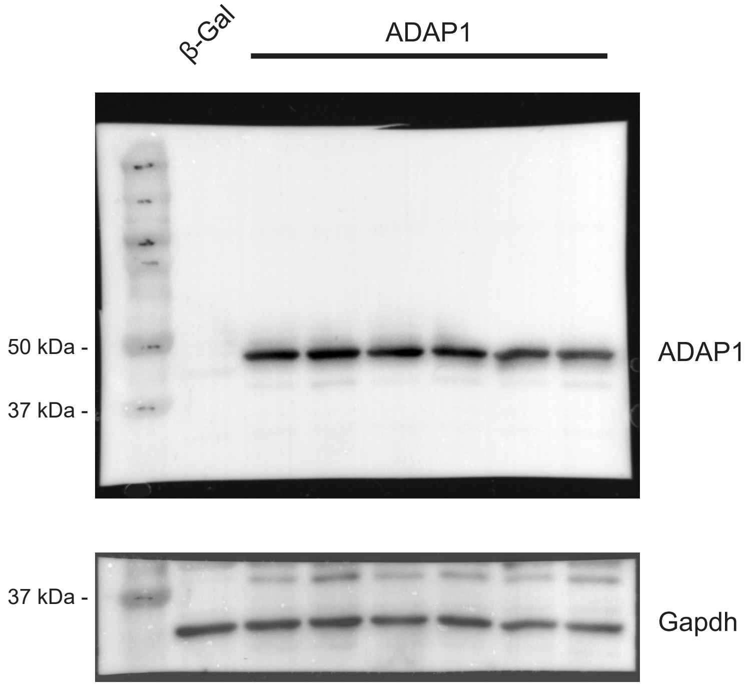

Supplementary Figure S2. ADAP1 and Gapdh full-length immunoblots

Supplementary Figure S3

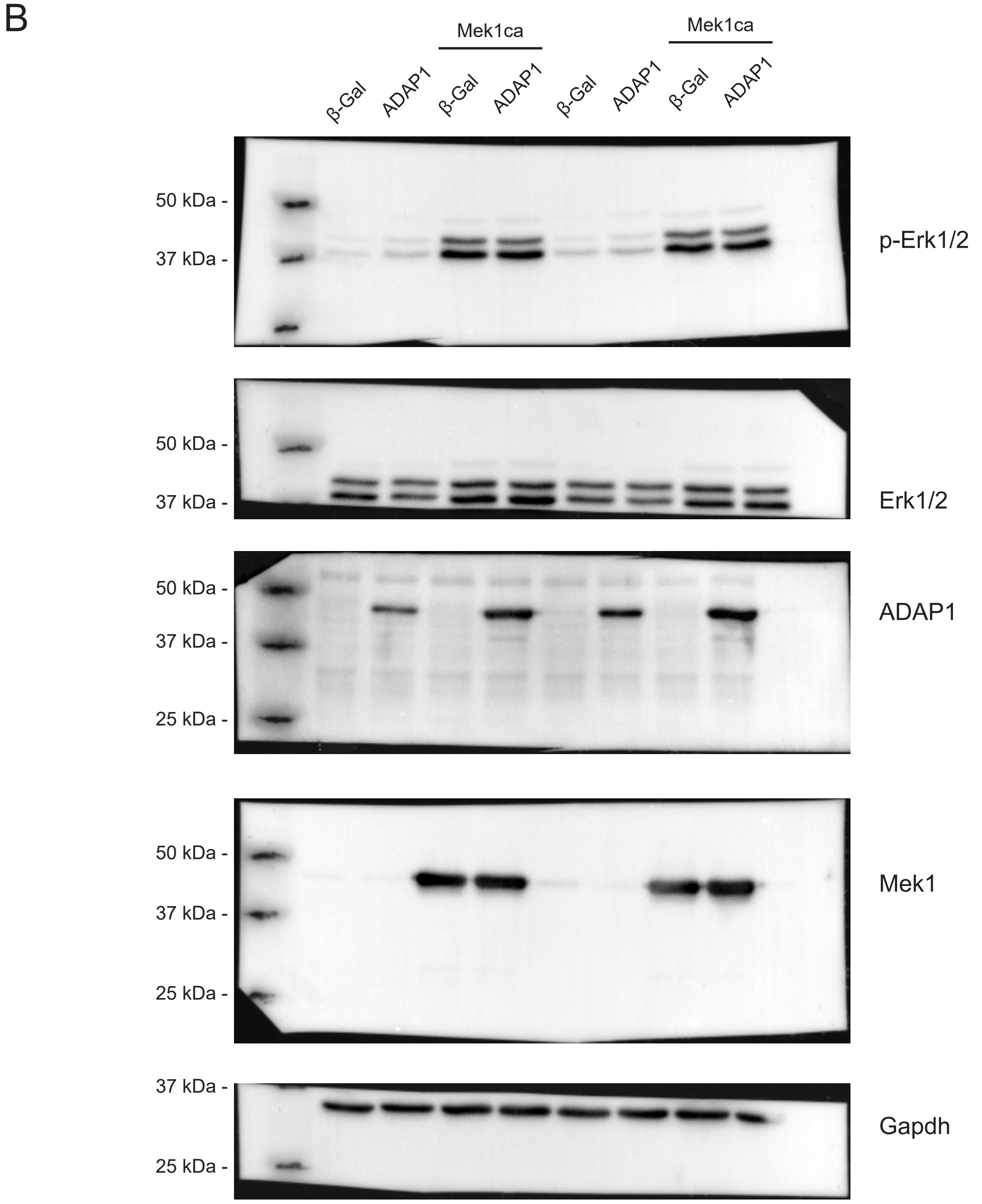

Supplementary Figure S3. p-Erk1/2, total Erk1/2, ADAP1, Mek1 and Gapdh full-length immunoblots

Supplementary Figure S6

A

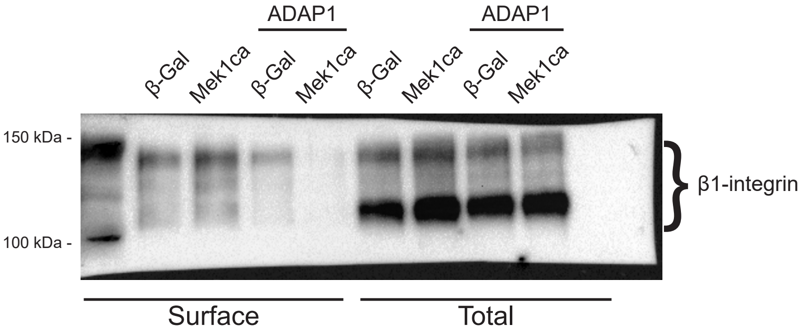

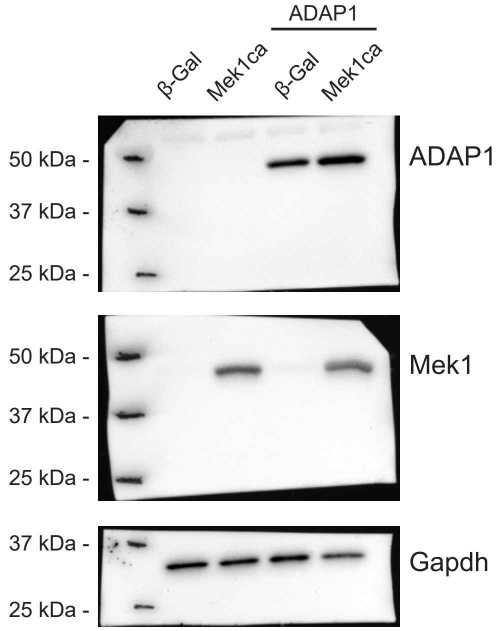

C

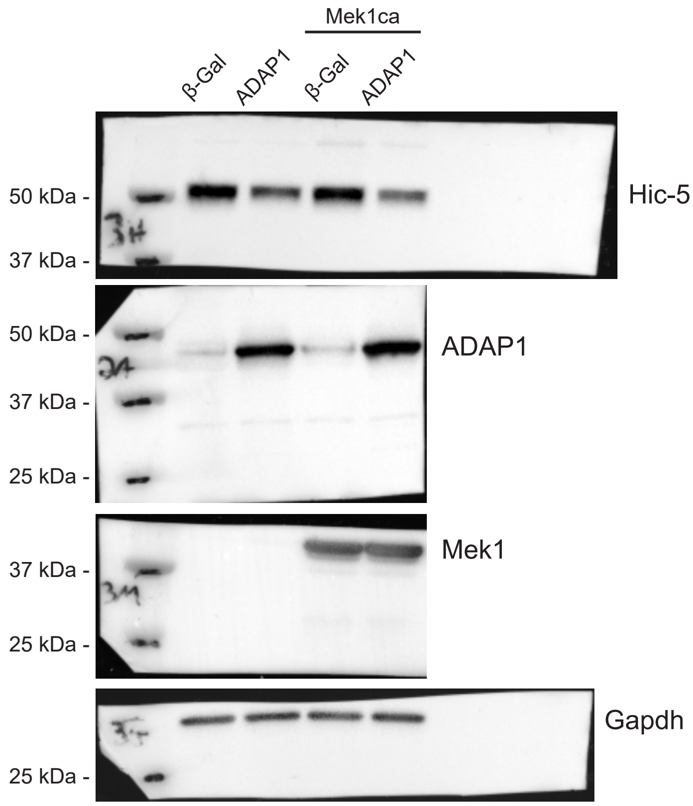

Supplementary Figure S6. β1-integrin, ADAP1, Mek1, Hic-5 and Gapdh full-length immunoblots
